# Gastrostomies: experience and complications with three modalities in a tertiary centre over a 26-year period

**DOI:** 10.3389/fmed.2023.1191204

**Published:** 2023-10-17

**Authors:** Ana Piñar-Gutiérrez, Pilar Serrano-Aguayo, Rocío Vázquez Gutiérrez, Silvia García Rey, Irene González-Navarro, Dolores Tatay-Domínguez, Pilar Garrancho-Domínguez, Pablo J. Remón-Ruiz, Antonio J. Martínez-Ortega, Verónica Nacarino Mejías, Álvaro Iglesias-López, María Socas, Salvador Morales-Conde, Francisco José García-Fernández, Juan Manuel Bozada-García, José Luis Pereira-Cunill, Pedro Pablo García-Luna

**Affiliations:** ^1^UGC Endocrinología y Nutrición, Hospital Universitario Virgen del Rocío, Sevilla, Spain; ^2^Servicio de Radiología, Hospital Universitario Virgen del Rocío, Sevilla, Spain; ^3^Servicio de Cirugía General, Hospital Universitario Virgen del Rocío, Sevilla, Spain; ^4^Servicio de Aparato Digestivo, Hospital Universitario Virgen del Rocío, Sevilla, Spain

**Keywords:** gastrostomy, enteral nutrition, complications, PEG, PRG, PLAG, interventional radiology

## Abstract

**Objectives:**

To describe the complications associated with the different gastrostomy techniques [endoscopic (PEG), radiologic (PRG), and surgical (SG)] performed in the last 26 years in a terciary hospital.

**Methods:**

Retrospective observational study. Patients who underwent gastrostomy at the Virgen del Rocío University Hospital between 1995 and 2021 were included. For PEG, the PULL technique was performed until 2018 and subsequently the PUSH technique predominantly. For PRG, a pigtail catheter was used until 2003, a balloon catheter between 2003 and 2009, and a balloon catheter with gastropexy between 2015 and 2021. For SG, the conventional technique (CSG) was performed until 2009 and since then the laparoscopic assisted percutaneous gastrostomy (PLAG) technique. Descriptive analysis was performed obtaining the median and quartiles of the quantitative variables [P50 (P25-P75)] and the frequency for the qualitative variables [*n* (%)].The comparison of complications between patients who underwent different techniques was performed with Fisher’s test.

**Results:**

*n* = 1,070 (PEG = 608, PRG = 344, SG = 118). The three most frequent indications were head and neck tumors, neurological diseases and gastroesophageal tumors. The percentage of patients who had any complication was 48.9% (PEG-PULL), 23.7% (PEG-PUSH), 38.5% (pigtail PRG), 39.2% (balloon PRG), 29.7% (balloon with gastropexy PRG), 87.3% (CSG), and 41.26% (PLAG). 2 (0.18%) patients died from gastrostomy-related complications. 18(1.68%) presented with peritonitis and 5 (0.4%) presented with gastrocolic fistula. The rest of the complications were minor.

**Conclusion:**

Gastrostomy in any of its modalities is currently a safe procedure with a low rate of complications, most of which are minor.

## 1. Introduction

Enteral nutrition by gastrostomy is the recommended option over nasogastric tube in patients requiring this support for a period longer than 4–6 weeks.

The first direct gastrostomy technique was described in 1898 by Stamn, and was performed by laparotomy with manual preparation of the stoma and suturing in a tobacco pouch (1). Subsequently, Janeway (1), in 1912, described a new surgical technique in which a valve was made with the gastric wall itself to prevent reflux of gastric contents, a frequent complication with Stamn’s technique. In 1980, Gauderer and Ponsky first described the percutaneous endoscopic gastrostomy (PEG) technique for paediatric patients (2), which was performed using the PULL technique. This technique achieved a reduction of complications and thus the generalisation of the gastrostomy technique for enteral nutrition or gastric decompression.

Since then, different techniques and variants of these techniques have emerged. The description in 1981 of the percutaneous radiological gastrostomy (PRG) technique by Preshaw (3) and in 1983 of the PUSH percutaneous gastrostomy technique or Sacks-Vine technique (4) stand out. Currently, both PEG and PRG can be performed with PUSH and PULL techniques.

Surgical techniques (SG) have also evolved. In addition to conventional direct techniques with manual ostoma preparation (Stamn and Janeway techniques), the percutaneous PUSH technique can be performed. All three techniques can currently be performed by laparotomy or laparoscopy. On the other hand, in our centre, and with the aim of reducing complications, we developed the percutaneous laparoscopic assisted gastrostomy (PLAG) technique, associated with a similar level of complications to PEG and PRG (5). The latter can also be performed by laparotomy if the patient is operated on by this route for another reason and this surgical procedure is used to perform a gastrostomy.

Currently, since percutaneous endoscopic and radiological techniques are associated with fewer complications than direct surgical techniques, their use is more widespread (6). Surgical techniques are therefore usually reserved for when it is not possible to perform any of them (because of anatomical anomalies such as the interposition of the colon or liver between the abdominal wall or stomach, because it is not possible to pass a nasogastric tube or endoscope, or because there is no transillumination) (5, 7).

The indications have also expanded and varied over time. Currently, the most frequent pathologies for which these techniques are indicated are head and neck cancer, neurological diseases, malignant and benign pathologies of the oesophagus and stomach, and maxillofacial pathologies (8).

Although these procedures have a low morbidity, they are not free of complications (9, 10), including minor (more frequent) complications such as exudate, irritation, granuloma, obstruction, tube leakage, etc.; and major (less frequent) complications such as aspiration, peritonitis and perforation, which can result in the death of the patient.

A large number of gastrostomies are performed at our tertiary hospital by teams with extensive experience in the three techniques. In addition, we have a nutrition unit, with specific consultations for the follow-up of these patients by a specialised nursing team and a medical team made up of specialists in endocrinology and nutrition, where a large volume of patients are seen. As technological improvements have become available, the number of complications in our series has declined (5, 11–14).

The primary objective of our study is to describe the complications associated with the different gastrostomy techniques used in the last 25 years in our centre. The secondary objectives are: to compare the complications associated with the new techniques with those associated with the techniques previously used and currently in disuse in our centre, and to describe the indications for these techniques as well as their evolution over this period.

## 2. Methods

### 2.1. Design

A retrospective observational study was carried out including all adult patients who had undergone gastrostomy by endoscopic, radiological or surgical technique at the Hospital Universitario Virgen del Rocío and who were subsequently followed up at the hospital’s Nutrition Unit between 1995 and 2021.

### 2.2. Data collection, variables, and monitoring

Data collection was carried out retrospectively using the clinical records of the Andalusian Health Service. Until around 2008–2009, medical records were collected on paper and subsequently began to be collected in Digital Medical Records. Written informed consent was obtained from all the patients before the procedure. All the required clinical and ethical guidelines of our center were followed.

The study variables included were: sex, age, date of performance, indication for gastrostomy (head and neck tumours, oesophageal tumours, non-tumoural oesophageal diseases, ALS, other neurological diseases, severe malabsorption, maxillofacial diseases and others), type of gastrostomy, presence of complications, presence of major complications (peritonitis, need for invasive mechanical ventilation (IMV) after the procedure and gastrocolic fistula), presence of minor complications (non-purulent exudate, irritation, burn due to gastric contents, balloon leakage, obstruction of the tube lumen, stoma dilatation, bleeding, granuloma, balloon rupture and/or local infection -as inflammation and purulent exudate with presence of microorganisms in the culture and need for antibiotic treatment-) and death due to gastrostomy complications. The follow-up period ranged from 3 to 24 months until the patient’s loss of follow-up in the Nutrition Unit.

### 2.3. Statistical analysis

Statistical analysis was carried out using Statistical Package for Social Science (SPSS®) 25 version for Windows (IBM Corporation, New York, USA). Descriptive analysis was performed by obtaining the median and quartiles for quantitative variables [expressed as P50 (P25-P75)] and the frequency for qualitative variables [expressed as *n* (%)]. Fisher’s test was used to analyze the differences between patients who underwent different techniques and their complications.

### 2.4. Techniques performed

The technique used in each patient was not randomised, but decided by a team specialised in Clinical Nutrition. SG was used in those cases in which PEG and PRG were not possible (due to complete obstruction of the upper gastrointestinal tract, lack of transillumination, some anatomical abnormalities and/or interposition of the colon or liver between the abdominal wall and the stomach).

- PEG: the PULL endoscopic technique (Ponsky-Gauderer technique) was performed exclusively until 2018, when the PUSH technique (Sacks-Vine technique) was introduced. The latter was the predominant technique in 2019 and 2021.
- PRG: until September 2003, only catheters with internal fixation of the pigtail type (Cope type) were used, then simple balloon catheters were used until 2009 (for simplicity it will be referred to as PUSH technique without pexy) and finally the PUSH technique with internal fixation with balloon and additional anchoring system with pexy (for simplicity it will be referred to as PUSH technique with pexy) was used from 2009 onwards.
- SG: conventional direct surgical techniques (Stamn or Janeway; CSG) were performed until 2010, when they began to be performed using the PLAG technique (described by our group in 2016 (5)).

Enteral nutrition was started 6 h after the procedure in the case of PEG and PRG techniques and 24 h in the case of SG, except in those cases where there was clinical suspicion of peritonitis. After admission, patients were assessed in Clinical Nutrition consultations at 1 month and every 3 months thereafter.

## 3. Results

A total of 1,070 patients were included. PEG was performed in 608 patients (515 PULL type, 93 PUSH type), PRG in 344 patients (114 pigtail type, 28 balloon type without pexy and 202 PUSH type with pexy) and SG in 118 patients (55 CSG and 63 PLAG).

The demographic characteristics of the patients (gender and age) are shown in Table 1.

**Table 1:** Sex and age distribution of patients who underwent gastrostomy at the Hospital Universitario Virgen del Rocío between 1995 and 2021.

	PEG		PRG			SG	
	PULL (1995–2018, *n* = 515)	PUSH (2018–2021, *n* = 93)	Pigtail (1995–2003, *n* = 114)	PUSH without pexy (2003–2009, *n* = 28)	PUSH with pexy (2009–2021, *n* = 202)	CSG (1995–2010, *n* = 55)	PLAG (2010–2021, *n* = 63)
Male sex	334(64,9%)	57(61,3%)	88(77,2%)	15(53,6%)	133(66%)	46(83,6%)	50(79,36%)
Age (years)	60(42–71)	64(55–73)	52(40–62)	63(52–74)	62(56–70)	60(50–67)	60(50–73)

The pathologies that led to the indication of gastrostomy in these patients are shown in Table 2.

**Table 2:** Pathologies that led to the indication of different gastrostomy techniques at the Hospital Universitario Virgen del Rocío between 1995 and 2021.

Technique Indication	PEG		PRG			SG	
	PULL (1995–2018, *n* = 515)	PUSH (2018–2021, *n* = 93)	Pigtail (1995–2003, *n* = 114)	PUSH without pexy (2003–2009, *n* = 28)	PUSH with pexy (2009–2021, *n* = 202)	CSG (1995–2010, *n* = 55)	PLAG (2010–2021, *n* = 63)
Head and neck cancer	153 (29,8%)	8(14,8%)	51(44,7%)	16(57,1%)	73(38,8%)	15(26,8%)	14(24,1%)
Esophagogastric tumour	8(1,5%)	7(12,9%)	13(11,4%)	3(10,7%)	21(11,1%)	22(39,3%)	19(32,8%)
Non-tumour oesophagogastric pathology	4(0,7%)	3(5,5%)	0(0%)	0(0%)	3(1,5%)	0(0%)	2(3,4%)
ALS	9(1,7%)	10(18,5%)	2(1,7%)	6(21,4%)	58(30,8%)	1(1,8%)	3(5,2%)
Neurological disease	281(54,8%)	24(44,4%)	42(36,8%)	1(3,5%)	26(13,8%)	12(21,4%)	17(29,3%)
Severe malabsorption	11(2,1%)	0(0%)	0(0%)	0(0%)	1(0,5%)	0(0%)	0(0%)
Maxillofacial pathology	1(0,1%)	0(0%)	3(2,6%)	0(0%)	1(0,5%)	0(0%)	0(0%)
Others	42(8,2%)	1(1,8%)	3(2,6%)	0(0%)	9(4,7%)	5(8,9%)	3(5,2%)

Complications associated with the PEG performed are shown in Table 3. 48.9% of patients with PULL PEG suffered some complication associated with the procedure, the most frequent being exudate (30.7%) followed by granuloma (22.3%). Complications occurred in 23.7% of patients with PEG PUSH and 2 (2.2%) had associated peritonitis. No patient died of peritonitis. Significant differences were found between groups in terms of total complications (*p* < 0.001), exudate (*p* < 0.001) and granuloma (*p* < 0.001), more frequent in the PEG PULL group and bleeding (*p* = 0.023), more frequent in the PEG PUSH group. All bleeding events were mild.

**Table 3:** Complications associated with the performance of PULL and PUSH PEG in 608 patients seen in the Nutrition Unit of the Hospital Universitario Virgen del Rocío between 1995 and 2021.

Complication	PEG PULL (1995–2018, *n* = 515)	PEG PUSH (2018–2021, *n* = 93)	*p*
Patients with any complication	252(48,9%)	22(23,7%)	<0.001
Death due to gastrostomy-related complications	0(0%)	0(0%)	
Major complications			
Peritonitis	7(1,4%)	2(2,2%)	0.634
Need for VMI after procedure	0(0%)	0(0%)	
Gastrocolic fistula	2(0,3%)	0(0%)	1
Minor complications			
Exudate	158(30,7%)	0(0%)	<0.001
Irritation	20(3,9%)	4(4,3%)	0.735
Burn	0(0%)	0(0%)	
Leakage	64(12,4%)	9(9,7%)	0.289
Obstruction	8(1,6%)	4(4,3%)	0.096
Stoma dilation	0(0%)	0(0%)	
Bleeding	0(0%)	2(2,2%)	0.023
Granuloma	115(22,3%)	6(6,5%)	<0.001
Breakage	15(2,9%)	3(3,2%)	0.538
Local infection	20(3,9%)	2(2,2%)	0.32

The complications associated with the different PRG techniques performed in our centre between 1995 and 2021 are shown in Table 4. It should be noted that no major complications were recorded with pigtail PRG. The percentage of patients with complications ranged from 29.7% (PUSH PRG with pexy) to 39.2% (PUSH PRG without pexy). The PUSH technique without pexy was associated with peritonitis in 25% of cases. One patient died in 2019 following PUSH type PRG with pexy in the context of peritonitis after dehiscence of the pexy suture, in addition to severe upper airway obstruction due to a well-differentiated pharyngeal carcinoma cT4N3Mx.

**Table 4:** Complications associated with radiological gastrostomy in 344 patients seen in the Nutrition Unit of the Virgen del Rocío University Hospital between 1995 and 2021.

Complication	Pigtail (1995–2003, *n* = 114)	PUSH without pexy (2003–2009, *n* = 28)	PUSH with pexy (2009–2021, *n* = 202)	*p*
Patients with any complication	44(38,5%)	11(39,2%)	60(29,7%)	0.213
Death due to gastrostomy-related complications	0(0%)	0(0%)	1(0,5%)	1
Major complications				
Peritonitis	0(0%)	7(25%)	1(0,5%)	<0.001
Need for VMI after procedure	0(0%)	0(0%)	0(0%)	
Gastrocolic fistula	0(0%)	0(0%)	2(1%)	1
Minor complications				
Exudate	30(26,3%)	2(7,1%)	27(13,4%)	0.005
Irritation	0(0%)	0(0%)	0(0%)	
Burn	0(0%)	0(0%)	0(0%)	
Leakage	8(7%)	2(7,1%)	14(6,9%)	1
Obstruction	2(1,7%)	2(7,1%)	9(4,5%)	0.246
Stoma dilation	0(0%)	1(3,5%)	3(1,5%)	0.188
Bleeding	0(0%)	1(3,5%)	1(0,5%)	0.156
Granuloma	17(14,9%)	1(3,5%)	24(11,9%)	0.282
Breakage	2(1,7%)	0(0%)	8(4%)	0.448
Local infection	0(0%)	1(3,5%)	11(5,4%)	0.022

Gastro-colic fistula has occurred in two cases (1%) with the PUSH type PRG technique with pexy. The first case was a 73-year-old man with a head and neck tumour who 10 months after the procedure presented with severe malnutrition and diarrhoea with similar characteristics to the enteral nutrition formula he was receiving. A CSG gastrostomy was performed to resolve the situation. The second was a 64-year-old male with a head and neck tumour who presented severe malnutrition, diarrhoea and polymicrobial bacteraemia (*S. aureus*, *K. aerogenes*, and *E. faecalis*) secondary to the gastrostomy tube migration fistula. He was treated with intravenous antibiotherapy (cefazolin and piperacillin/tazobactam) and a PLAG gastrostomy was performed.

Complications associated with the SG techniques used are shown in Table 5. A complication occurred in 87.3% of patients who underwent CSG compared to 41.26% of patients who underwent PLAG (*p* < 0.001). In the former, the most frequent complication was irritation (81.8%) and in the latter, exudate (23.8%; *p* < 0.001). There were also statistical differences regarding exudate and burn (both were more frequent in the CSG group). A 23-year-old patient with a diagnosis of ALS presented with acute respiratory failure after the procedure in 2019. He required non-invasive mechanical ventilation and a 10-day ICU admission. Another 45-year-old patient with an oesophageal carcinoma died after balloon rupture and leakage of gastric contents leading to diffuse peritonitis with hepatic abscesses.

**Table 5:** Complications associated with the performance of surgical gastrostomy in 118 patients seen in the Nutrition Unit of the Virgen del Rocío University Hospital between 1995 and 2021.

Complication	CSG (1995–2009, *n* = 55)	PLAG (2010–2021, *n* = 63)	*p*
Patients with any complication	48(87,3%)	26(41,26%)	<0.001
Death due to gastrostomy-related complications	0(0%)	1(1,5%)	1
Major complications			
Peritonitis	0(%)	1(1,5%)	0.283
Need for VMI after procedure	0(%)	1(1,5%)	0.534
Gastrocolic fistula	0(%)	1(1,5%)	0.534
Minor complications			
Exudate	23(41,8%)	15(23,8%)	0.029
Irritation	45(81,8%)	8(12,7%)	<0.001
Burn	10(18,2%)	1(1,5%)	0.002
Leakage	4(7,3%)	7(11,1%)	0.348
Obstruction	0(%)	0(0%)	
Stoma dilation	3(5,5%)	1(1,5%)	0.26
Bleeding	1(1,8%)	1(1,5%)	0.717
Granuloma	6(10,9%)	9(14,2%)	0.395
Breakage	0(%)	1(1,5%)	0.534
Local infection	1(1,8%)	3(4,7%)	0.551

## 4. Discussion

The present study evaluates the rate of complications associated with the performance of 1,070 gastrostomies for enteral nutritional support in adult patients with different PEG, PRG and SG techniques employed over the last 25 years. The techniques were performed in a tertiary hospital by experienced staff and the patients were closely followed up in an experienced Nutrition Unit. In summary, the techniques that have been implemented over the years have decreased the percentage of associated complications, and these have been mostly minor, so they could be considered as safe techniques.

The most frequent indication for PEG was neurological diseases followed by head and neck tumours, as described in other studies (1, 2). For PRGs, the most frequent indication was head and neck tumours followed by neurological diseases. This is due to the fact that some patients with head and neck tumours have tumours that impede the passage of the endoscope, and has been described by other authors (3, 4). In these cases, the European endoscopy guidelines recommend that, in the event that PEG is performed, it should be of the PUSH type (5). In both techniques, there has been an increase in the number of patients who have undergone gastrostomy for ALS. This is due to the implementation of a specific multidisciplinary unit with the participation of specialists in clinical nutrition, which has led to an improvement in the care of these patients. The most frequent indication for SG was oesophagogastric tumours, followed by head and neck tumours, with no major changes between the two techniques and therefore between the two periods. These indications are consistent with the previously described reasons for deciding to perform SG instead of PEG or PRG in our centre.

Comparison of complications with other studies is complex due to the lack of uniformity in the definition of complications and the variants of the techniques themselves. Regarding PEG and PRG, our results are comparable to those described in the study by Galaski et al. (6). This study included patients seen between 2010 and 2015, so we should compare their results with our PEG PULL and PRG PUSH with pexy techniques. They obtained 50% of episodes of minor complications with the endoscopic technique and 36.3% with the radiological technique. Specifically, in that study there were fewer episodes of exudate in patients with PEG (16.7%) but a similar number with PRG (11.4%). Their infection rate in PEG (6.7%) was higher than ours, but similar in PRG (4.5%). Regarding tube exit, their rate was lower (6.7 and 4.5%), but they had more bleeding episodes (13.3 and 6.8%). Regarding major complications, they had a higher rate than ours (4%). Another multicentre study from Korea (2) in which 418 patients were included also reported some of the complications described in our study, with similar percentages for infection and obstruction, but differences in terms of exudate (0.9 and 3.2% with PEG and PRG respectively) and tube leakage (5.9 and 16%).

Still on complications associated with PEG and PRG, our data differ from those obtained in the study by Kohli et al., in which the total complications encountered were lower (7). This is due to methodological differences between the two studies, as this multicentre study only studied complications arising in the first 30 days after the procedure, whereas our study analyses complications arising in a long-term follow-up of the patient. In addition, this US study was conducted with a national database of readmissions using coded diagnoses, whereas in our study each history was reviewed individually.

Additionally, prospective multicenter studies and meta-analyses have also been performed, such as that of Grant et al. (8), although this was performed only in patients with head and neck cancer. In this study, mortality after PEG was 2.2% and after PRG 1.8%. Moreover, major complications occurred in 7.4 and 8.9% respectively, higher data than ours.

Regarding surgical techniques, there are few studies evaluating the new laparoscopic techniques and their long-term complications, but the PLAG technique has been shown to be safe (9, 10). In addition, the laparoscopically assisted percutaneous endoscopic endoscopic gastrostomy (LAPEG) technique, which has similar characteristics, has also demonstrated a low rate of associated complications (11, 12), even when compared to conventional PEG (13), especially in paediatric populations (14).

It should be noted that our results show that the appearance of new techniques (PUSH in PEG, PUSH with pexy in PRG and PLAG in SG) has led to a decrease in complications, as previously described in the scientific literature both by our group (9, 15, 16) and by other authors (17–20), although not without controversy in the case of both PEG techniques (21). Given the results, we can confirm that gastrostomy in any of its modalities is currently a safe procedure with a low rate of complications, most of which are minor. To all this must be added that a 2019 systematic review showed that the use of gastrostomy improves the patient’s quality of life (22).

These safety data are consistent with the results of the meta-analysis by Strijobs et al. (23) in which they compared PEGs with PRGs and found that there was no difference in mortality and major complications, and that these were rare. It also agrees with a meta-analysis comparing both groups but in patients with motor neuron disease. This last study also showed that PRG had higher success rates in this group of patients (24). If we want to compare both techniques, two studies of interest have recently been published. In 2022, a meta-analysis with GRADE study did show lower mortality, peritonitis and colon perforation in patients with PEG (25), and in 2023 also another meta-analysis appeared to demonstrate lower 30-day mortality, tube leakage, and tube dislodgement rates with the endoscopic technique (26).

It is worth mentioning that caution should be exercised with regard to the higher rate of bleeding (even if slight) with the recently implanted PEG PUSH technique. Precisely in the Endoscopic management of enteral tubes in adult patients guidelines of the European Society of Gastrointestinal Endoscopy (ESGE), it is considered a relative contraindication recent gastrointestinal (GI) bleeding due to peptic ulcer disease (5). In addition, these guidelines recommend the PULL technique as the standard technique for performing PEG, reserving the PUSH technique for cases in which it is not possible (strong recommendation, low quality evidence). However, the characteristics of each center should be taken into account, since it is precisely in our hospital that the PUSH technique is currently performed more frequently because the endoscopists have more training in this technique and are more comfortable with it. Our data on fewer complications reinforce this decision (27).

The main limitation of this study, in addition to its observational nature and retrospective data collection, is the heterogeneity of the patients included in terms of the techniques performed and the types and calibres of tube used. On the other hand, it would have been interesting to take into account some variables that were not collected and that may affect the number of complications: use of prophylactic antibiotic therapy (which has been shown to reduce the incidence of local infection in meta-analyses in patients with PEG (28)), use of anticoagulants, diameter of gastrostomy tubes, duration of procedures, follow-up time, nutritional status of the patient, nutritional formulas used and comorbidities of the patients. Collection was not possible as the oldest records are not in digital format and these data were not present in the records.

The inclusion in the study of patients with different pathologies and indications may represent another bias and sub-analyses should be performed in the future in these groups, since complications may vary among them and the indication for one technique or another may depend on this, and the choice of technique should be made in the most individualised manner possible as recommended in guidelines (5). Finally, the results of our study may not be extrapolated to other hospitals, as ours is a tertiary hospital with a higher level of resources, in which there are multidisciplinary units where less frequent pathologies are treated and the indications may differ from other centres.

## 5. Conclusion

We present a series of patients who underwent gastrostomy by endoscopic, radiological or surgical technique in a tertiary hospital over the last 26 years. The indication for gastrostomy has varied over this period. Gastrostomy in any of its modalities is currently a safe procedure with a low rate of complications, most of which are minor. However, patients should be carefully selected, the modality of gastrostomy should be discussed in a multidisciplinary way, experienced endoscopists, radiologists, and surgeons should be available, and any major complication should be analysed from a risk management perspective. Further studies, especially randomised clinical trials that analyse complications based on each indication, will allow us in the future to individualise the choice of the most appropriate technique for each patient.

## Data availability statement

The raw data supporting the conclusions of this article will be made available by the authors, without undue reservation.

## Ethics statement

The studies involving human participants were reviewed and approved by CEI de los Hospitales Universitarios Virgen Macarena – Virgen del Rocío de Sevilla. The patients/participants provided their written informed consent to participate in this study.

## Author contributions

AP-G and PG-L: conceptualization. SG and RV: methodology. PG-L: software, resources, supervision. PS-A, JP-C, and PG-L: validation. AP-G: formal analysis, writing—original draft preparation, visualization. AP-G, IG-N, DT-D, PG-D, PR-R, and AM-O: investigation. SG, RV, and AP-G: data curation. AP-G, JP-C, MS, SM-C, JB-G, FG-F, and PG-L: writing—review and editing. ÁI-L, VN, and PG-L: project administration. ÁI-L and PG-L: funding acquisition. All authors contributed to the article and approved the submitted version.
